# Position paper: a coordinated approach to fracture and fall prevention from seven international organizations

**DOI:** 10.1007/s41999-026-01596-7

**Published:** 2026-09-10

**Authors:** Nathalie van der Velde, Lotta J. Seppala, Gulistan Bahat, Hubert Blain, Alvaro Casas Herrero, Nicholas C. Harvey, Tahir Masud, René Rizzoli, Jean-Yves Reginster, Carmelinda Ruggiero, Mario Barbagallo, Alex Barreto de Lima, Maria Bonnici, Jean Bousquet, Bernard Cortet, Lorenzo Chiari, Yannis Dionyssiotis, Karsten Dreinhöfer, Gustavo Duque, Chris Easthope Awai, Frede Frihagen, James Frith, Ellen Freiberger, Deniz Cengiz, Eleftheria Giannouli, Tomasz Grodzicki, Nataliia Grygorieva, Evrim Gökçe, Jorunn L. Helbostad, Christof J. Kadane, Meltem Koca, Marko Kolšek, Tomasz Kostka, Jūratė Macijauskienė, Finbarr C. Martin, Lisa McGarrigle, Emeline Michel, Marta Neira Álvarez, Maria Panourgia, Stany Perkisas, Christoph Pertinatsch, Carmen Queirós, Regina Roller-Wirnsberger, Erik Rosendahl, Jesper Ryg, Cheryl A. Sadowski, Opinder Sahota, Schroder Sattar, Dawn A. Skelton, François-Xavier Sibille, Balamrit Singh Sokhal, Joke Spildooren, Emma Stanmore, Timo E. Strandberg, Tim Stuckenschneider, Chris Todd, Hana Vankova, Ellen Vlaeyen, Ozlem Yilmaz, Yelda Öztürk

**Affiliations:** 1 Internal Medicine, Section of Geriatric Medicine, UMC Location University of Amsterdam, Meibergdreef 9, Amsterdam, The Netherlands; 2 Amsterdam Public Health, Aging and Later Life, Amsterdam, The Netherlands; 3 Department of Internal Medicine, Division of Geriatrics Capa, Istanbul Medical Faculty, Istanbul University, Istanbul, Turkey; 4 Department of Geriatrics, Montpellier University Hospital, MUSE, Montpellier, France; 5 Geriatrics Department, Universitario de Navarra (HUN), C/Irunlarrea S/N 31008, Pamplona, Spain; 6 Navarra Institute for Health Research (IdiSNA), Pamplona, Spain; 7 CIBER of Frailty and Healthy Aging (CIBERFES), Instituto de Salud Carlos III, Madrid, Spain; 8 MRC Lifecourse Epidemiology Centre, University of Southampton, Southampton, UK; 9 NIHR Southampton Biomedical Research Centre, University of Southampton and University Hospital Southampton NHS Foundation Trust, Southampton, UK; 10 International Osteoporosis Foundation, Nyon, Switzerland; 11 Department of Geriatric Medicine, Nottingham University Hospitals NHS Trust, Nottingham, UK; 12 Geneva University Hospitals and Faculty of Medicine, Geneva, Switzerland; 13 Protein Research Chair, Biochemistry Dept, College of Science, King Saud University, Riyadh, Kingdom of Saudi Arabia; 14 Orthogeriatric and Geriatric Units, Gerontology and Geriatrics Section, Department of Medicine and Surgery, University of Perugia, S. Maria Misericordia Hospital, S. Andrea Delle Fratte, 06132 Perugia, Italy; 15 Department of Internal Medicine, University of Palermo, Palermo, Italy; 16 Department of Internal Medicine and Geriatrics, Geriatric Unit, University of Palermo, Palermo, Italy; 17 Physical Education Course, Nilton Lins University, Amazonas, Brazil; 18 Department of Geriatric Medicine, Karin Grech Rehabilitation Hospital, Pieta`, Malta; 19 Mater Dei Hospital L-Imsida, Malta & St Vincent De Paul, Luqa, Malta; 20 Allergy Department, Charité Hospital, Berlin, Germany; 21 MASK-Air SAS, Montpellier, France; 22 Department of Rheumatology, University-Hospital of Lille, 59000 Lille, France; 23 Department of Electrical, Electronic, and Information Engineering “Guglielmo Marconi”, Alma Mater Studiorum-Università di Bologna, Bologna, Italy; 24 Physical Medicine and Rehabilitation Department, National Rehabilitation Center EKA, Athens, Greece; 25 Department of Orthopedics, Charité University Medicine, Medical Park Berlin Humboldtmühle, Berlin, Germany; 26 Division of Geriatric Medicine, Department of Medicine, McGill University, Montreal, QC Canada; 27 Data Analytics & Rehab Technology (DART), Lake Lucerne Institute, Rubistrasse 9, 6354 Vitznau, Switzerland; 28 Østfold Hopsital Trust, Kalnes, Norway; 29 Institute of Clinical Medicine, University of Oslo, Oslo, Norway; 30 Falls and Syncope Service, The Newcastle Upon Tyne Hospitals NHS Trust, Newcastle, UK; 31 Institute for Biomedicine of Aging FAU Erlangen-Nürnberg, Nuremberg, Germany; 32 Department of Senior Health, St. George’s University Hospitals NHS Foundation Trust, London, SW17 0QT UK; 33 Department of Internal Medicine Division of Geriatric Medicine, Hacettepe University, Ankara, Turkey; 34 Rehabilitation Engineering Laboratory, Department of Health Sciences and Technology, ETH Zurich, Zurich, Switzerland; 35 Department of Internal Medicine and Geriatrics, University Hospital, Jagiellonian University Medical College, Cracow, Poland; 36 Department of Clinical Physiology and Pathology of Locomotor Apparatus, D.F. Chebotarev Institute of Gerontology, NAMS of Ukraine, Kiev, Ukraine; 37 Comete Laboratory, INSERM UMR-S 1075, UNICAEN, Caen, France; 38 Department of Neuromedicine and Movement Science, The Norwegian University of Science and Technology, Trondheim, Norway; 39 Department of Medicine, St. Olav University Hospital, Trondheim, Norway; 40 Department for Scientific Research in Rehabilitation, Pension Insurance Austria, Vienna, Austria; 41 Research Unit for Aging and Life Long Health, Medical University of Graz, Graz, Austria; 42 Department of Geriatric Medicine, Etlik City Hospital, 06170 Yenimahalle Ankara, Turkey; 43 Health Center Litija and Department of Family Medicine, Medical Faculty, University of Ljubljana, Ljubljana, Slovenia; 44 Department of Geriatrics, Medical University of Lodz, Lodz, Poland; 45 Department of Geriatrics, Lithuanian University of Health Sciences, Kaunas, Lithuania; 46 Faculty of Life Sciences and Medicine, Population Health Sciences, King’s College London, London, UK; 47 Faculty of Science and Health, Atlantic Technological University, Co. Mayo, Atlantic, Ireland; 48 Acute Geriatric and Therapeutic Care Unit, University Côte d’Azur, Nice University Hospital, Nice, France; 49 Geriatrics Department, University Hospital of Infanta Leonor, Madrid, Spain; 50 Care of the Elderly Department, Milton Keynes University Hospital, Milton Keynes, UK; 51 University Center for Geriatrics, ZAS / University of Antwerp, Antwerp, Belgium; 52 Nursing School, University of Porto, Porto, Portugal; 53 RISE-Health, Porto, Portugal; 54 Health Sciences Research Unit: Nursing (UICISA:E), Nursing School of Coimbra, Coimbra, Portugal; 55 Department of Internal Medicine Research Unit for Aging and Life Long Health, Medical University of Graz, Graz, Austria; 56 Department of Community Medicine and Rehabilitation, Umeå University, Umeå, Sweden; 57 Department of Clinical Medicine, University of Copenhagen, Copenhagen, Denmark; 58 Department of Internal Medicine Geriatric Section, Geriatric Research and Clinical Evidence (GRACE), Copenhagen University Hospital - Herlev and Gentofte Hospital, Copenhagen, Denmark; 59 Faculty of Pharmacy & Pharmaceutical Sciences, University of Alberta, Edmonton, AB Canada; 60 Department of Geriatric Medicine, Nottingham University Hospitals NHS Trust, Nottingham, UK; 61 College of Nursing, University of Saskatchewan, Saskatoon, Canada; 62 Research Centre for Health (ReaCH), Glasgow Caledonian University, Glasgow, UK; 63 Geriatric Medicine Departement, CHU UCL Namur, Yvoir, Belgium; 64 Institute of Health and Society and Clinical Pharmacy and Pharmacoepidemiology Research Group, Louvain Drug Research Institute, Université Catholique de Louvain, Brussels, Belgium; 65 School of Medicine, Keele University, Keele, Staffordshire UK; 66 University Hospital of North Midlands, Stoke-On-Trent, Staffordshire, UK; 67 REVAL Rehabilitation Research Center, Hasselt University, 3590 Diepenbeek, Belgium; 68 School of Health Sciences, The University of Manchester, Manchester, UK; 69 Manchester University NHS Foundation Trust, Manchester, UK; 70 University of Helsinki and Helsinki University Hospital, Helsinki, Finland; 71 The Finnish Osteoporosis Association, Helsinki, Finland; 72 Department for Health Services Research, Geriatric Medicine/ School of Medicine and Health Services, Carl von Ossietzky University, Oldenburg, Lower Saxony Germany; 73 Department of Internal Medicine, Third Faculty of Medicine, Cooperatio34, Charles University and University Hospital Královské Vinohrady, Prague, Czech Republic; 74 Faculty of Medicine and Life Sciences, Research Group Healthcare & Ethics, UHasselt, 3590 Diepenbeek, Agoralaan Belgium; 75 Faculty of Medicine, Department of Public Health and Primary Care, Academic Centre for Nursing and Midwifery, KU Leuven, Kapucijnenvoer 7, bus 7001, 3000 Louvain, Belgium; 76 Department of Internal Medicine, Division of Geriatrics, Health Science University Istanbul Training and Research Hospital, Istanbul, Turkey; 77 Department of Geriaric Medicine, Etlik City Hospital, 06170 Yenimahalle Ankara, Turkey

**Keywords:** Falls, Fragility fracture, Older people, Osteoporosis

## Abstract

Fragility fractures constitute a major global health burden and often result from falls. However, clinical care for fragility fractures is currently organized separately from fall prevention services leading to gaps in integrated care delivery. This joint position paper from the European Geriatric Medicine Society, the Fragility Fracture Network, the World Falls Prevention Society, the European Society for Clinical and Economic Aspects of Osteoporosis, Osteoarthritis and Musculoskeletal Diseases, the International Osteoporosis Foundation, the European Union of Medical Specialists-Geriatric Medicine Section, and the International Association of Gerontology and Geriatrics–European Region highlights the critical need for integrated falls and fracture prevention, with a focus on evidence-based clinical practice, knowledge dissemination, education, policy strategies, and research. We outline existing guidelines on fall and fracture prevention, with particular emphasis on how fracture risk assessment can be incorporated into fall prevention services and on integrating fall assessment into fracture prevention services. We advocate that future implementation guidelines place greater emphasis on fully integrated models of care. Moreover, training and education programs should be designed to encourage interdisciplinary collaboration among healthcare professionals. Integrated fall and fracture prevention services should be recognized as a public health priority and incorporated into national strategies for healthy aging, supported by adequate, dedicated, and sustainable resources including evidence-based exercise provision. Finally, future research should focus on evaluating the (cost) effectiveness of integrated care models and examining their practical implementation in real-world settings.

**Supplementary Information:**

The online version contains supplementary material available at https://doi.org/10.1007/s41999-026-01596-7.

## Introduction

Fragility fractures pose a major public health challenge in Europe, contributing substantially to immobility, loss of independence, increased morbidity, mortality, and reduced quality of life [1]. Fragility fractures occur particularly in adults over 50 years, where low bone mineral density (BMD) contributes substantially to fracture risk [2]. A well-characterized example of a fragility fracture is at the hip, which is associated with substantial morbidity and 1-year mortality of 20–24% [3]. Functional recovery among survivors is limited, with 40% unable to ambulate independently and 60% still requiring assistance 1 year after the injury [3]. Consequently, approximately one-third of patients become fully dependent or require residential care within the year following a hip fracture [3]. In 2021, the International Osteoporosis Foundation (IOF) introduced an updated report of the scorecard for osteoporosis in Europe (SCOPE) for the EU27, along with Switzerland and the UK [4]. In 2019, an estimated 4.3 million new fragility fractures occurred in this region, including approximately 827,000 hip fractures. The direct cost of new fractures was estimated at €36.3 billion, with an additional €19.0 billion attributable to long-term disability from fractures sustained in previous years. Pharmacological assessment and treatment accounted for a further €1.6 billion, bringing the total direct cost in this region of Europe during 2019 to €56.9 billion. Healthcare costs remain higher than the pre-fracture level for the five years following the fracture [5].

**Fig. 1:**
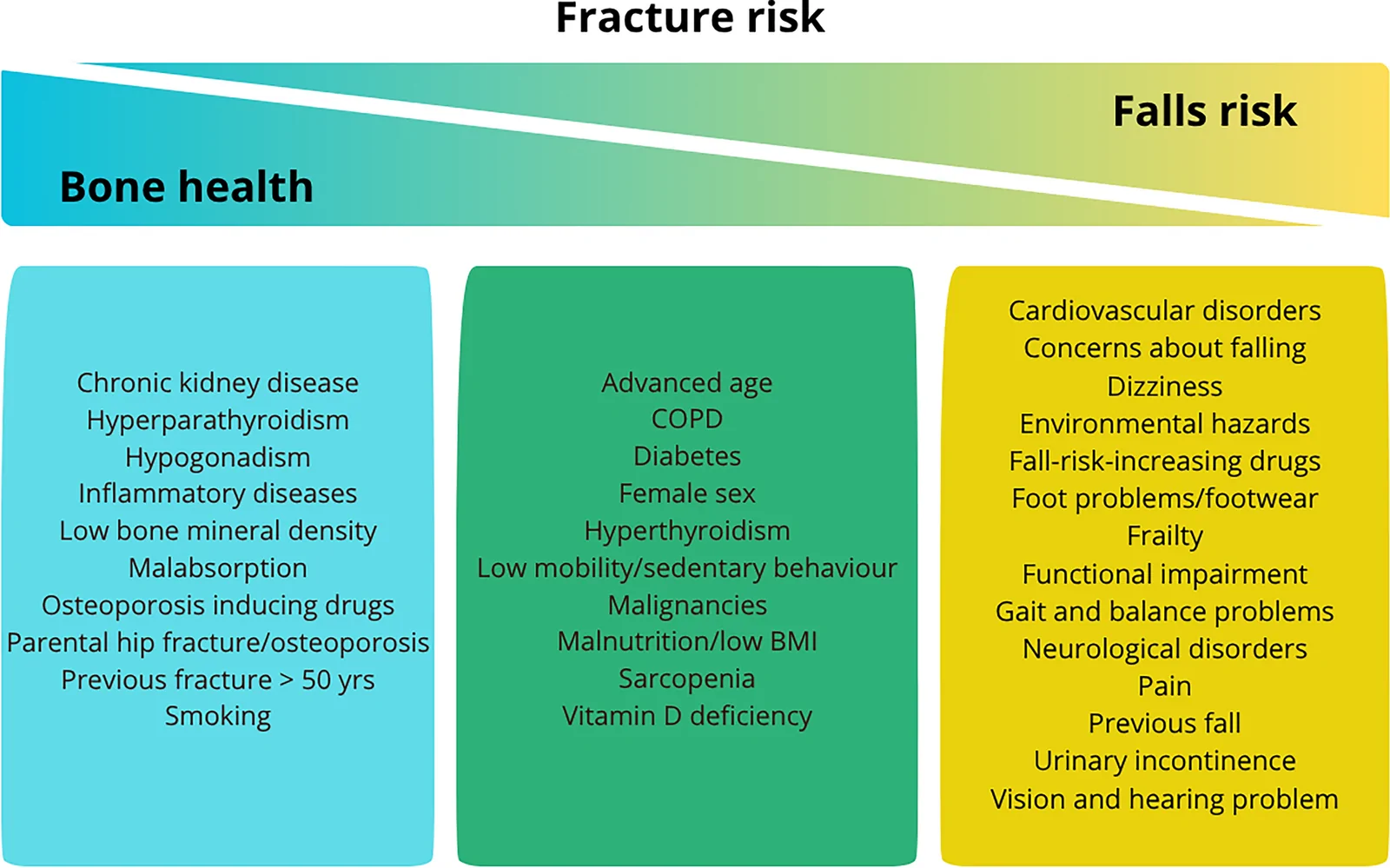
Comprehensive fracture risk assessment incorporating both bone health–specific and fall-related risks factors

Fracture prevention is closely linked to fall prevention. More than 95% of hip fractures are caused by falling [3]. Fig. 1 presents a conceptual framework illustrating how bone-related factors and fall risk together contribute to fracture risk. The relative contribution of bone fragility and falls to the risk of non-vertebral fractures changes over time. Furthermore, osteosarcopenia, defined as the combination of sarcopenia and osteopenia/osteoporosis, is a common geriatric syndrome among older adults with an estimated prevalence ranging from 5 to 37% among community-dwelling older adults and is associated with risk of falls and fractures [6]. In addition, several studies have shown an imminent subsequent fracture risk after both incident fractures as well as incident falls [7–10]. Similarly, an increased risk of falling soon after fracture has also been demonstrated [11–13]. Thus, given the interconnected risk of falls and fractures, a coordinated and comprehensive approach to fall and fracture prevention is warranted. However, fracture prevention has historically focused on increasing BMD, often without incorporating a concurrent clinical approach to fall prevention. Conversely, fall prevention strategies have typically failed to adequately address bone health. Despite clear bidirectional associations between falls and fractures, service delivery, clinical pathways, and research agendas remain largely siloed. This fragmentation represents a missed opportunity to reduce morbidity, mortality, and healthcare expenditure.

In 2016, the European Geriatric Medicine Society (EuGMS) Special Interest Group on Falls and Fracture prevention in collaboration with the International Association of Gerontology and Geriatrics for the European Region (IAGG-ER), the European Union of Medical Specialists Geriatric Medicine Section (UEMS), and the International Osteoporosis Foundation–European Society for Clinical and Economic Aspects of Osteoporosis and Osteoarthritis (IOF-ESCEO) published a position paper that provided a comprehensive overview of fracture risk factors and outlined prevention strategies, emphasizing the importance of a dual target managing bone health alongside fall risk to address this lack of integration [14]. Since then, the evidence base has evolved substantially, notably with the release of the 2022 World Guidelines for Fall Prevention and Management (WFG), which presented robust, evidence-based recommendations for fall prevention [15]. Parallel advances have also been made in fracture prevention and management, with updated guidelines for management of osteoporosis in women [16] and men from IOF and ESCEO [17], the global IOF Capture the Fracture initiative launched by the IOF [18], a clinical toolkit released in 2020 by the Fragility Fracture Network (FFN) [19], the introduction of therapeutic approaches guided by baseline fracture risk from ESCEO and IOF[20] [21], and entry of several new osteoporosis medications to the market [22].

In response, the EuGMS Special Interest Group on Falls and Fracture Prevention, in collaboration with the Fragility Fracture Network (FFN), the World Fall prevention Society (WFPS), IOF, ESCEO, UEMS, and IAGG-ER convened to update and expand the original position paper. Supplementary Table 1 presents an overview of these organizations**.** The recommendations presented in this position paper are based on a review of existing literature, complemented by expert knowledge. The paper was developed by a dedicated writing committee (NvdV, LS, GB, HB, ACH, TM, NH, RR, JYR, CR) that convened an initial meeting to define the primary aims and scope of the manuscript. NvdV and LS prepared the initial draft. Subsequently, the recommendations and content were reviewed and refined with input from the entire writing committee and, following that, from a multidisciplinary expert group comprising 50 authors from 23 countries, all with expertise in fall and fracture prevention and management. The aim of this position paper is to emphasize the importance of integrating fall and fracture prevention, rather than proposing a new guideline. Our objective is to align existing evidence and recommendations into a coherent, operational framework that supports implementation in real-world clinical settings.

### Clinical practice

We refer to the existing published guidelines and other resources on fall and fracture prevention, as outlined in Table 1**,** to support clinical practice. Effective fracture prevention requires a dual approach: (1) osteoporosis management, and (2) comprehensive fall prevention and management. Although existing guidelines acknowledge the importance of fracture prevention within fall prevention strategies and refer readers to fracture prevention guidelines, and vice versa, these approaches often remain fragmented. There is a clear need to develop integrated guidelines in the future that explicitly connect fall prevention and fracture prevention, along with enhanced interprofessional collaboration between the organizations responsible for their development. Delivering such integration requires purposeful clinical leadership to align aims across organizations, foster interprofessional teamwork, and steward implementation at scale. In this context, emerging digital technologies, such as remote monitoring, wearable sensors, and data-driven risk stratification tools, may support more continuous, personalized, and coordinated care pathways, facilitating the integration of fall and fracture prevention within routine clinical practice. Given often limited healthcare resources, effective fall and fracture prevention also depends on collaboration between healthcare professionals, local communities, patient organizations, and other relevant stakeholders to enhance reach and scalability.

**Table 1:** Key clinical resources to support fall and fracture prevention

Fall prevention and management
World guidelines for fall prevention and management for older adults (WFG): a global initiative [15]
Fall prevention in community-dwelling older adults and implementation of world falls guidelines: a call for action across Europe by the European Geriatric Medicine Society Special Interest Group on Falls and Fractures [23]
WHO: Step Safely: Strategies for preventing and managing falls across the life-course [24]
National guidelines on fall prevention such as the NICE guideline: Falls: assessment and prevention in older people and in people 50 and over at higher risk [25]
**Fracture prevention and management**
FFN clinical toolkit supporting the prevention and management of fragility fractures. including pillars: (i) multidisciplinary co-management of the acute fracture episode, (ii) optimizing rehabilitation to recover function, independence and quality of life, and (iii) reliable delivery of secondary fracture prevention after every fragility fracture [19]
Capture the Fracture® global program providing recognition, resources, training and tools to support post-fracture care coordination programs (Fracture Liaison Services) worldwide [26]
**Osteoporosis diagnosis and management**
European guidance for the diagnosis and management of osteoporosis in postmenopausal women [16]
Efficacy of osteoporosis pharmacological treatments in men: a systematic review and meta-analysis [27]
Evidence-based guideline for the management of osteoporosis in men [17]
Treatment of Osteoporosis and Osteoarthritis in the Oldest Old [28]
Personalized therapy stratified by baseline fracture risk [20, 21]
National guidelines on osteoporosis such as the NOGG UK clinical guideline [29]
Strong, steady and straight: UK consensus statement on physical activity and exercise for osteoporosis [30]

**Fig. 2:**
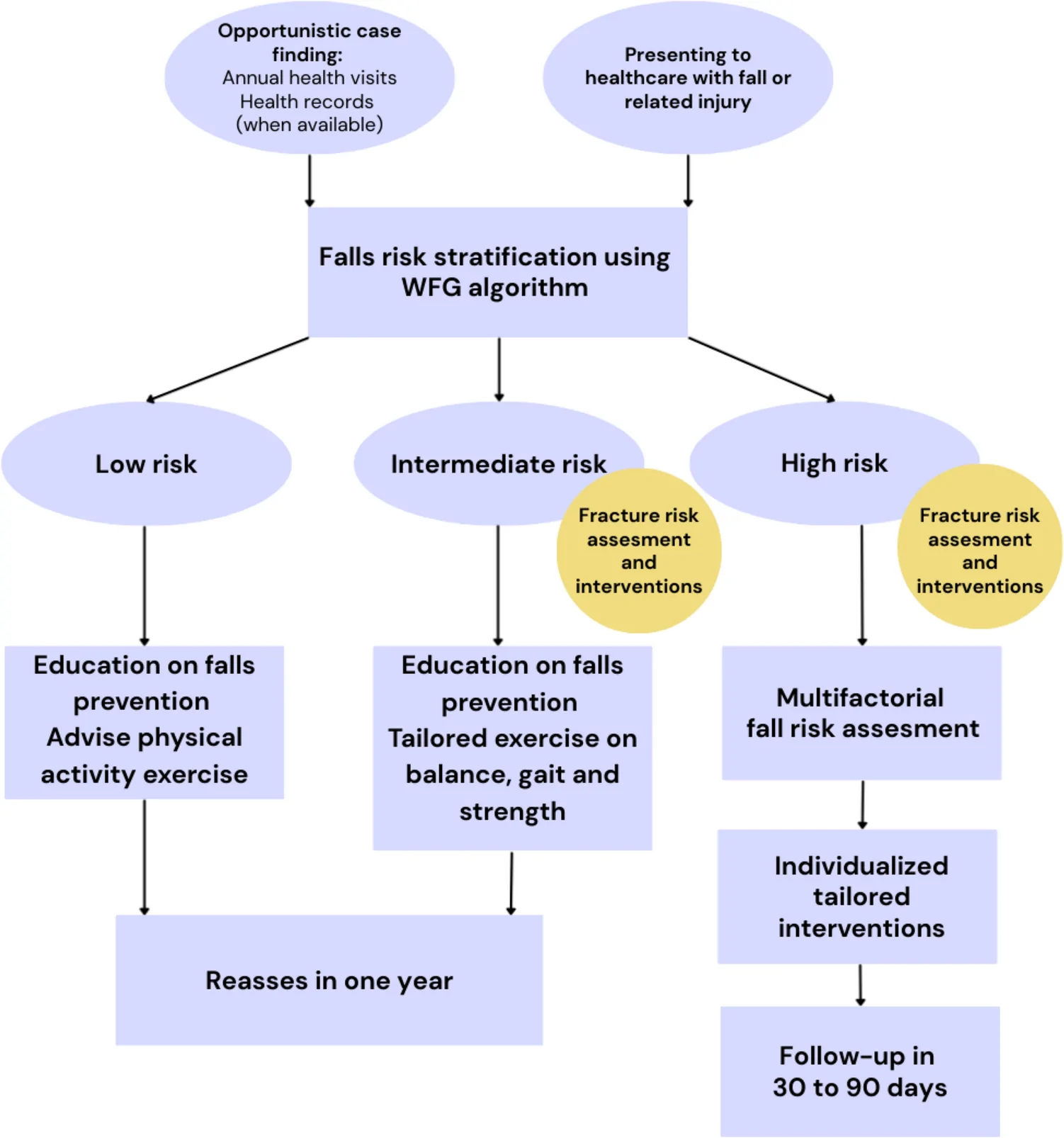
Fracture risk assessment based on World Falls Guidelines [15]

**Fig. 3:**
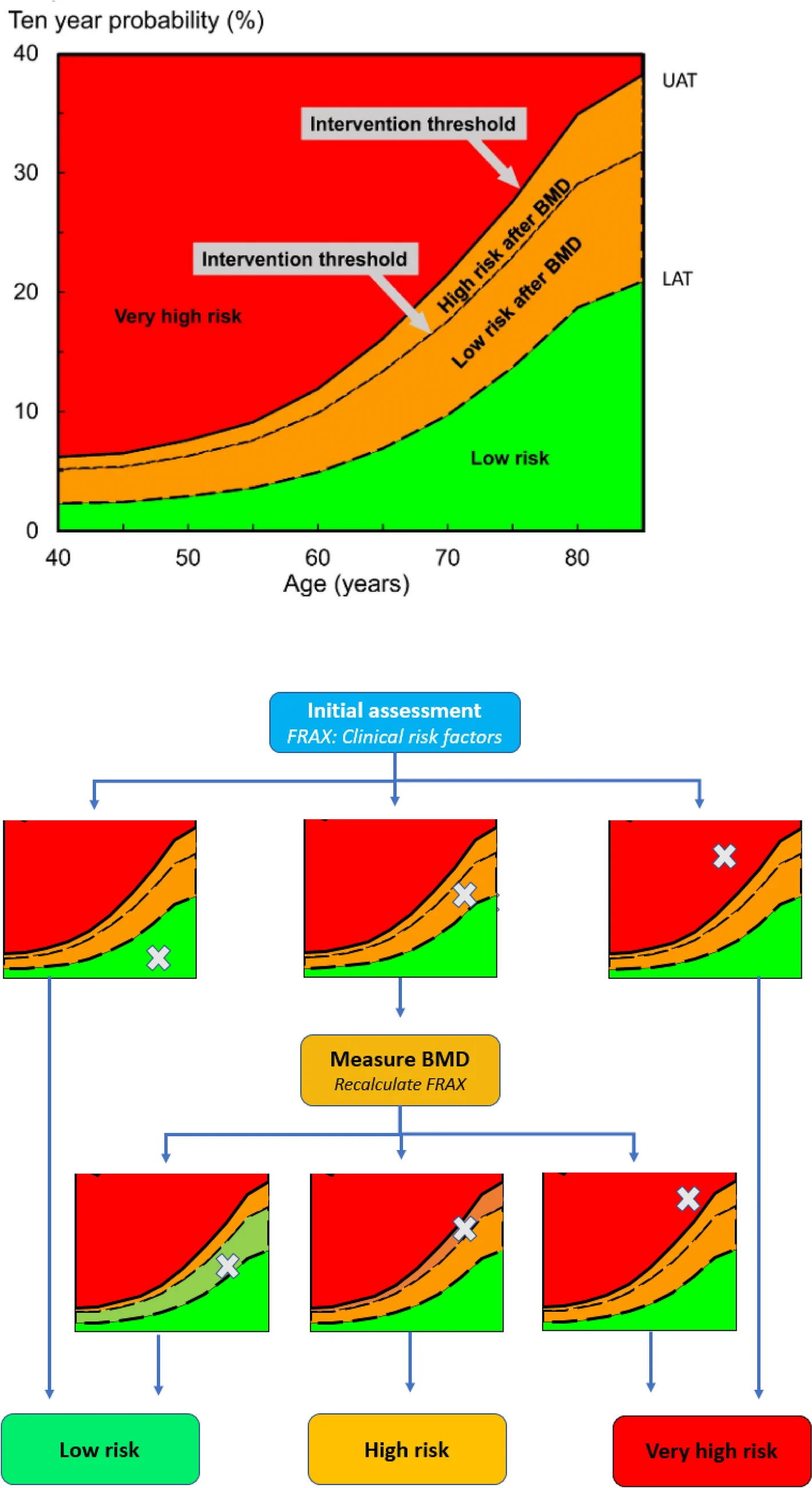
The characterization of fracture risk by FRAX major osteoporotic fracture probability in postmenopausal women. Originally published in [20]

Currently, there is considerable room for improvement in integrating fracture prevention within fall prevention services and care pathways. In a recent survey among healthcare professionals, fewer than 60% of the respondents reported including fracture risk assessment often or always in the multifactorial fall risk assessment [31]. In 2022, the WFG introduced a novel fall risk stratification algorithm for community-dwelling older adults to differentiate individuals at high risk of fall from low- and intermediate-risk older persons (https://worldfallsguidelines.com/algorithm) [15]. The WFG further recommends that those identified as having a moderate to high risk of fall should have a bone health assessment using local protocols (Fig. 2). According to the WFG, fracture risk assessment tools such as FRAX®, Garvan, and QFracture (in the UK) can be employed to identify older adults at high fracture risk, and bone densitometry can be used to confirm osteoporosis. In clinical practice, fall history should be regarded as a modifiable fracture risk amplifier and should directly inform both risk stratification and therapeutic choice. Garvan and QFracture include falls as a predictor in their calculation; FRAXplus® facilitates modification of a probability result derived from conventional FRAX® estimates of probabilities of hip fracture and major osteoporotic fracture with the knowledge of number of falls in the previous year [32, 33]. Kanis et al. developed an international algorithm for assessing fracture risk in postmenopausal women [20] **(**Fig. 3**).** Initial fracture risk assessment in postmenopausal women is estimated using FRAX based on clinical risk factors, with risk categorized into green (low), orange (intermediate), or red (very high) zones. Intermediate risk requires BMD testing and FRAX recalculation. After recalculation, risk may be in the red zone (very high risk), orange zone (high risk) or green zone (low risk, either in the original green zone or in the original orange zone but below the intervention threshold). Women with a prior fragility fracture are automatically considered at least high risk. The use of BMD testing varies across countries [16]. Furthermore, in a variety of other osteoporosis treatment guidelines, falls are identified as a risk factor for stratifying patients into higher fracture risk categories and thereby a more potent treatment choice [34, 35].

**Fig. 4:**
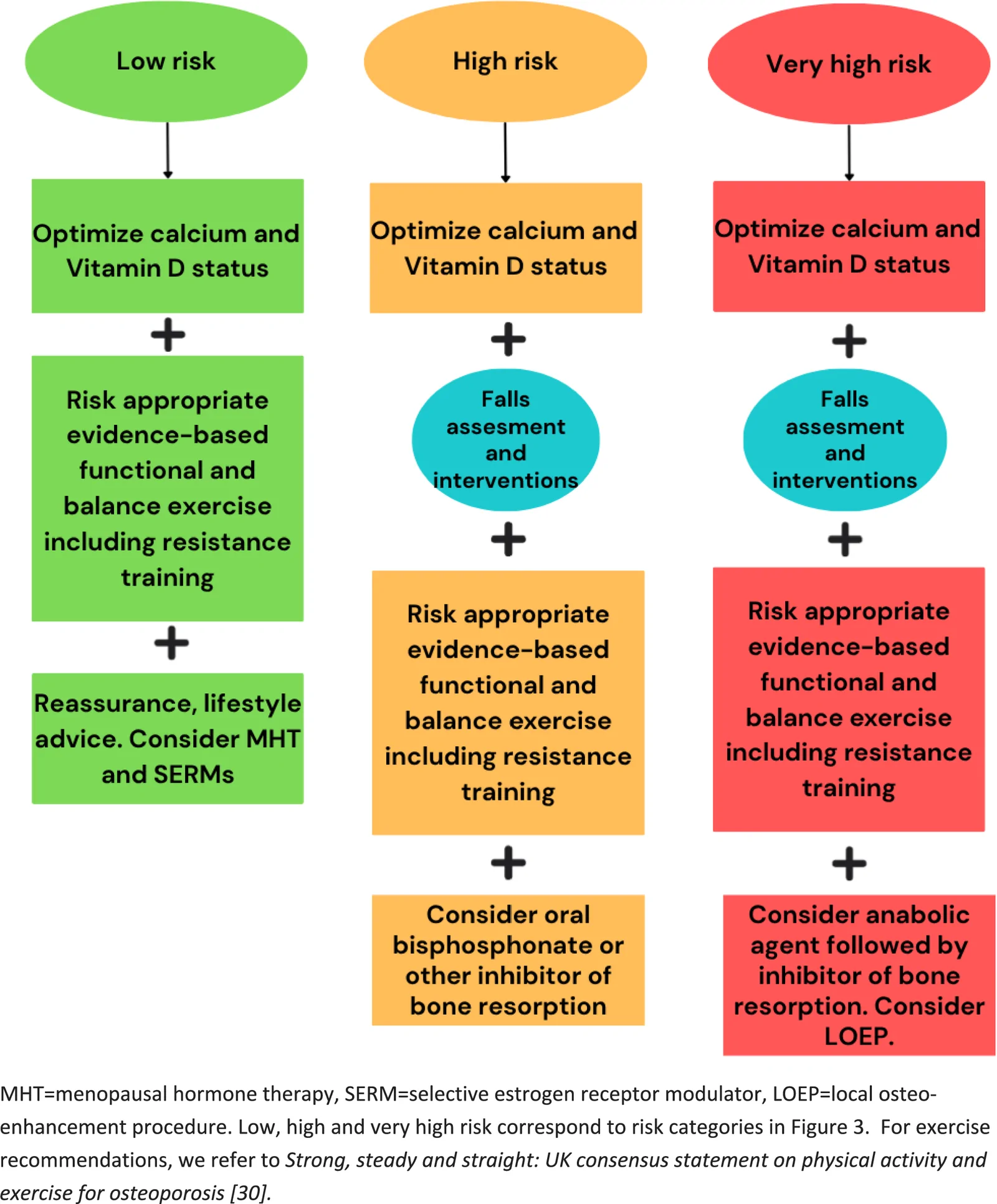
Treatment pathways and fall assessment/management according to the categorization of fracture risk. Adapted from [20]

On the other hand, fall prevention should be an integral component of fracture prevention, as emphasized by guidelines. For example, Kanis et al. (2020) presented an internationally applicable algorithm for managing postmenopausal women at low, high, and very high risk of osteoporotic fractures [20]. Importantly, they recommend that fall prevention strategies be embedded within the treatment pathway for patients at high and very high fracture risk (Fig. 4**)**.

More than half of patients suffering a hip fracture live with frailty [36]. Frailty describes a vulnerable state of older persons and is characterized by an increased susceptibility to stressors and an elevated risk of adverse outcomes such as fractures, falls, and all-cause mortality [37]. Managing frailty of hip fracture patients requires specialized expertise that can be ensured through the implementation of orthogeriatric care. Orthogeriatric care is a medical–surgical model combining multidisciplinary health professionals and has been proven effective in hip fracture care, lowering the number of consultations, complications, and readmissions and increasing bone health assessment and mobility. The FFN Orthogeriatric Care Model combines emergency, pre-operative, operative, and post-operative phases, as well as rehabilitation and return to function, and includes both fall and fracture prevention. In addition, in many countries, secondary prevention of fractures is organized through Fracture Liaison Services (FLS), in which fall risk assessment and intervention are recommended components. However, fall risk assessment is not routinely performed in many FLS settings, and referrals to fall risk intervention programs are infrequent. Overall, in a 2025 national UK FLS evaluation, approximately 65% of patients received or were referred for a fall risk assessment, with a large variation between FLS services [38], and this is probably lower or even absent in other countries [39] demonstrating that better integration between fall and fracture services is warranted. For example, in the Czech Republic, the need to refer patients to fall prevention and frailty intervention programs is mentioned in the national guidelines for osteoporosis [40] and in the statement on the introduction of FLS [41], while this plan has not yet been implemented and fall prevention is not yet provided for FLS participants according to published evidence [42]. Furthermore, there is often a lack of clear, practical guidance on fall assessments and interventions at FLS in the guidelines. For example, Capture the Fracture® advises that fall prevention at FLS should align with local, regional, and national guidelines and be conducted in those presenting with fragility fractures [18]. While most fall services target individuals aged 65 and older, a basic assessment is also necessary for those aged 50–64, with high-risk individuals to be referred to specialized services. This represents a critical gap, as FLS programs are uniquely positioned to operationalize integrated care, but frequently remain predominantly bone focused rather than comprehensively risk focused. Fig. 4 illustrates how fall assessment could be operationalized within FLS for patients with fractures, considering the heterogeneity of the FLS patient population.

**Fig. 5:**
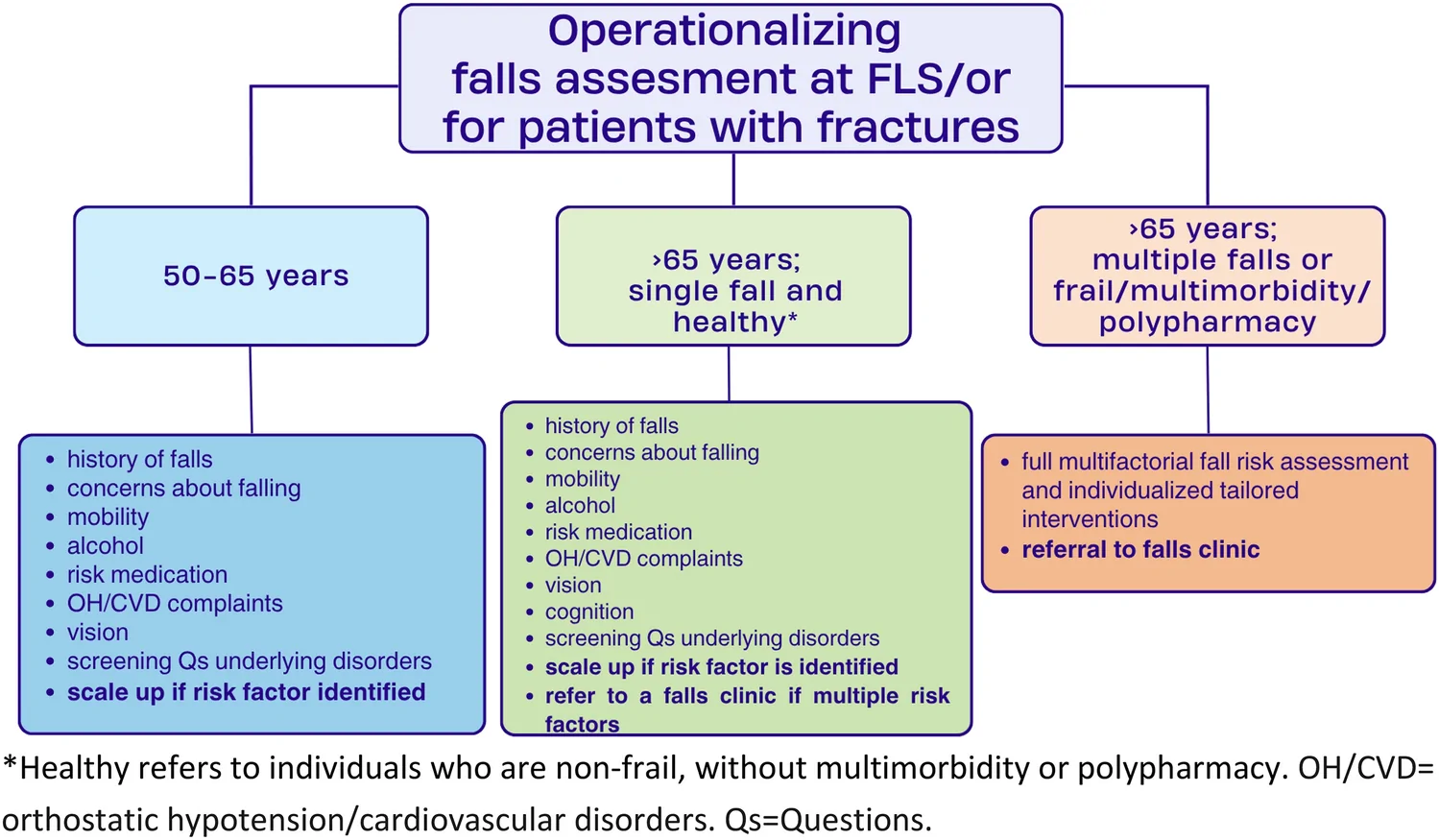
Operationalizing falls assessment at FLS/or for patients with fragility fractures

Domains for assessment and measurement approach according to WFG [15]: Concerns about falling:. Assess concerns about falling (previously called fear of falling), preferably using a validated instrument, for example by Falls Efficacy Scale-International (FES-I) or Short-FES-I. Mobility: i) Balance: Screen for balance disorders, for example by tandem stand, one Leg stand. If indicated, perform a full assessment in a structured manner, for example, by the Berg Balance Scale, POMA (subscale balance), Mini-BEST test. Gait: Assess both qualitatively and quantitatively using preferred speed 4-m walking length (< 0.8 m/s), POMA (subscale gait), dual task test, and functional gait assessment. Screen for mobility problems using a structured approach, for example, by Short Physical Performance Battery (SPPB), Timed Up and Go (TUG), Get Up and Go, including qualitative assessment. Muscle strength: Screen quantitatively using, for example, the chair stand test or hand grip strength. If indicated, assess structurally specific muscle groups (MRC scale). Risk medication: Perform a structured medication review that entails considering deprescribing of psychotropic, cardiovascular, and other FRIDs, for example, by applying STOPPFall, STOPP, and START or STEADI instruments. OH/CVD (orthostatic hypotension/cardiovascular disorders) complaints: i) OH: Measure blood pressure first supine (after a minimum of 5 min of bed rest) and repeatedly upon standing. Preferably continuously, or alternatively at 1-min intervals up to a minimal of 3 min and optimally 5 min, check for symptom recognition. ii) CVD: Assess by focused history taking about cardiovascular symptoms, history of cardiovascular disease, focused cardiovascular physical examination, and 12-lead surface electrocardiogram. If indicated, further assessment (may include tilt table testing, including carotid sinus massage, ambulatory rhythm monitoring, and/or blood pressure monitoring). Vision: Assess subjective vision problems (history taking). Objective assessment of visual problems and acuity, and appropriate use of glasses (including check multi−/bifocal glasses). Screening questions underlying disorder: Perform a clinical geriatric assessment (history taking, physical examination, laboratory measurements, additional testing when indicated) with specific attention toward diabetes mellitus, osteoarthritis, neurological disorders including PD, polyneuropathy and stroke, cardiovascular diseases, cognition, depressive disorders, delirium, anemia, electrolyte disorders, thyroid disease, frailty, and sarcopenia. Assess for potential atypical disease presentation of acute conditions such as pneumonia, especially in an acute care setting. Fig. 5.

Still, in many European countries, dedicated fall prevention services and FLS remain absent or insufficiently implemented, and access to orthogeriatric care remains limited or non-existing. These gaps have been highlighted, for example, in studies describing the current state of care in Türkiye [43, 44]), which also propose potential solutions to address these shortcomings. This underscores the need to map the baseline situation across countries and to develop resource-aware clinical and implementation guidelines for integrated falls and fracture care that prioritize the most effective, scalable interventions and can be adapted to local conditions. Even in countries considered to have well-developed fall and fracture prevention services implementation of evidence-based programs continues to be challenging and requires consistent approaches to facilitate implementation and ensure fidelity of delivery [45, 46].

Furthermore, a specific population suffering from falls and fractures are people with dementia. Approximately, 60–80% of people with dementia fall annually [47]. This is due to multiple factors including impaired mobility, impaired judgement, medications, mental health problems, and environmental hazard [48]. Moreover, the estimated prevalence of dementia in older adults with hip fractures is approximately 19%, whereas cognitive impairment is present in approximately 40% of all older adults with hip fractures [49]. Therefore, the WFG recommends that i) routine assessment of cognition should be included as part of multifactorial fall risk assessment in older adults, ii) community-dwelling older adults with mild cognitive impairment and mild to moderate dementia should be offered an exercise program to prevent falls, and iii) both older adults and caregiver’s perspective should be included when creating fall prevention care plans for adults with cognitive impairment [15]. Moreover, cognitively impaired and dementia patients should not be denied fracture prevention measures including pharmacological treatments for osteoporosis.

### Education

A recent survey conducted as part of the European Cooperation in Science and Technology (COST) Action 21,122, PROmoting GeRiAtric Medicine in countries where it is still eMergING (PROGRAMMING), included responses from healthcare professionals working in acute, rehabilitation, long-term care, and community settings [50]. Among the 3,922 respondents, approximately 12% reported low or very low knowledge of both falls and bone health, and 35.9% reported low knowledge of orthogeriatric care. Another recent survey conducted by the EuGMS SIG Falls and Fractures revealed widespread dissatisfaction with undergraduate education in fall prevention among all healthcare professionals, with only about a quarter of respondents agreeing or strongly agreeing that it adequately prepared them for clinical practice [31]. Encouragingly, the PROGRAMMING survey also demonstrated a high level of interest among respondents in falls, bone health, and orthogeriatric care.

In 2014, the European undergraduate curriculum in geriatric medicine was published, defining the minimum requirements that medical students should acquire before graduating [51]. The curriculum states students should be able to describe the pathophysiology, diagnosis, assessment, management, and preventive strategies for common geriatric syndromes in older adults, including falls and movement disorders. They should also be able to describe relevant aspects of pathophysiology, diagnosis, management and preventative strategies for common problems in older adults, such as osteoporosis. However, it is unclear whether these topics are taught separately in the undergraduate curriculum or integrated within a cohesive framework across Europe. In 2025, new Education Training Requirements (ETR) for geriatric medicine, which aim at setting uniform standards of knowledge, skills, and competencies for all postgraduate training, were adopted by the European Union of Medical Specialists (UEMS) [52]. The topic of falls and fractures is covered within ETR; however, the extent to which they are incorporated into postgraduate training as an interconnected topic depends on the education providers and may vary across countries, depending on the local policy and healthcare systems. A thorough understanding of the current approach is warranted not only in the medical profession but across all relevant professions, as these topics should be taught within an integrated, cohesive framework rather than as siloed subjects.

Furthermore, postgraduate training initiatives that integrate fall and fracture prevention for all healthcare professionals (HCPs) across different settings are important, as most HCPs, regardless of their specialty, will inevitably care for older patients. Ideally, such training should be interprofessional, fostering collaboration among medical, physiotherapy, nursing, pharmacy, dietitians, and other allied health professionals to provide coordinated, person-centered care. Although a range of local, national, and international organizations have developed educational initiatives, often focusing on either fall or fracture prevention, closer collaboration is needed to create truly integrated educational initiatives. Furthermore, defining a core set of competencies for integrated fall and fracture assessment would provide a common foundation for training healthcare professionals, while allowing local healthcare teams to adapt and implement programs according to their available resources, while remaining true to the evidence base.

### Policy

Considering the substantial evidence on the burden of falls and fragility fractures and the existence of effective interventions, integrated fall and fracture prevention should be recognized as a public health priority, supported by dedicated funding across Europe and globally. Beyond recognition, measurable implementation targets and accountability mechanisms are required to translate strategy into sustained practice change. Reflecting on the WHO’s Decade of Healthy Ageing (2021–2030), fall and fracture prevention should be explicitly embedded into national healthy aging strategies as a core intervention to preserve intrinsic capacity and prevent functional decline in older adults. Despite the strong evidence base and multiple existing reports, limited governmental engagement and persistent challenges in achieving political prioritization have frequently been noted [53]. In a competitive policy environment, reversing decades of policy stagnation is undoubtedly a challenge [53]. To address this, the FFN released a policy toolkit in 2020 to guide and support advocacy efforts on the prevention of fragility fractures [53]. Furthermore, one of the five pillars of the Capture the Fracture® Partnership is policy, with the main deliverable being a visualization of the impact of fragility fractures and the benefits of secondary fracture prevention at the policy level to inform changes in supporting FLS implementation [54]. A key element of the policy resources is the description of the anticipated clinical benefits and budget impact of secondary prevention as governments are motivated to realize cost reductions. This information is then combined with current and upcoming policy initiatives to guide recommendations and implementation strategies for policy changes at both national and regional levels. Furthermore, the WHO and the ESCEO have signed a 5-year collaboration agreement to improve bone health and prevent fragility fractures, particularly in older adults. The partnership aims to develop a strategic global roadmap on bone health and aging and to advocate for strong public health strategies to reduce fractures and strengthen fracture care services worldwide [55].

There are also some promising national strategies in place. For example, a national plan was launched in France in 2022 with the aim of reducing fractures and deaths related to falls by 20% [56]. This plan includes easier access to fall risk assessments by healthcare professionals according to the WFG. The national plan aims to facilitate access to recommended measures for people at moderate or high risk. The development of specific local consultations on ‘Balance and fall prevention’ is planned, as well as fall clinics that will interface with local FLS. In the UK policy focus on fall and fracture prevention has centered around the work of the National Fall Prevention Co-ordinating Group (NFPCG), originally set up by Public Health England providing guidance on fall and fracture prevention [57]. Furthermore, the Dutch government has introduced the "Integrated Approach to Fall Prevention" strategy, with support from the Dutch Injury Prevention Center (VeiligheidNL) [58] This approach includes identifying those at increased risk, assessing causes, offering proven exercise programs to improve strength and balance, and guiding older adults toward long-term physical activity to stay independent and prevent injuries. Interprofessional expert support is crucial when designing these strategies. Another example highlighting that dedicated expertise is essential for successful implementation is the Flanders Center of Expertise for Falls and Fracture Prevention in Belgium [59]. Effective system-level leadership is needed to align stakeholders, establish shared accountability, and support continuous quality improvement alongside the resources and KPIs required for integrated services. In addition, active involvement with older adults’ organizations and patient representative groups is essential to ensure that policies and interventions reflect the needs and preferences of the target population.

Many countries have established national hip fracture registries or audit systems; however, a substantial number still lack such infrastructure. Hip fracture registries provide important benefits, including monitoring the quality of care, benchmarking outcomes across institutions, identifying gaps in service delivery, and informing policy and quality-improvement initiatives. The FFN has released a minimal dataset for hip fracture registries [60]. While the elements include information on bone health medication, no variable related to fall prevention has been included. Given the importance of integration of fall and fracture prevention, the inclusion of an item documenting whether the participants have received fall preventive measures should be discussed and considered in future revisions of the dataset. Some countries have already incorporated fall prevention into their hip fracture database. For example, the Danish National Hip Fracture Database has included fall prevention since 2010 as one of the quality markers [61]. In England and Wales, the NHS funds an integrated national falls and fractures audit program (including the national hip fracture database) provided by the Royal College of Physicians of London and this encourages direct linkage between incident hip fracture management and secondary falls and fracture prevention [62].

## Research

There is a clear need for more integrated research in the field of fall and fracture prevention as well as the development of a coordinated research agenda. For example, multifactorial fall prevention trials have rarely included bone health as a key component in their assessments [63]. Emerging evidence suggests that integrated approaches to fall and fracture prevention show promising results. In an Australian pre–post study, the implementation of a novel care model with a multifactorial focus on both falls and fracture prevention was associated with reductions in incident falls, physiological falls risk, incident fractures, and FRAX-calculated fracture risk [64]. However, there remains a need to confirm the generalizability of the effectiveness and cost-effectiveness of integrated fall and fracture prevention strategies across clinical settings and populations. Implementation science will be essential to understand how such integrated models can be translated, adapted, and sustained across real-world healthcare settings. In light of the recent European position paper on fall prevention, which highlights the need to update to the ProFaNE Core Outcome Set for fall prevention [23], it is important to evaluate whether fractures should be included in this update. It would be beneficial to understand the extent to which different fall prevention programs reduce fracture risk.

Furthermore, reported phenotypic characteristics of FLS populations vary widely across studies in terms of mean age, proportion of men, and fracture location [65]. Similarly, the prevalence of osteoporosis, vertebral fractures, newly identified contributors to secondary osteoporosis or metabolic bone disease, and fall-related risk factors differ substantially between cohorts [65]. Given the lack of clear guidance on organizing fall prevention strategies for this heterogeneous group, further research is needed to assess fall risk across the lifespan, from age 50 years to the oldest old, following a first fracture, and to evaluate the effectiveness of preventive interventions in this population.

Additionally, a narrative review by Voltan et al. examined whether anti-osteoporosis medications can reduce falls, focusing on pharmacological interventions that show potential in lowering fall incidence in osteoporotic patients [66]. The authors concluded that further studies are needed in which fall risk is a primary outcome and physical functioning is assessed. Mockel et al. showed in their meta-analysis that romosozumab has a tendency to reduce the risk of falls in postmenopausal women with osteoporosis [67]. In their discussion, the authors highlighted the need for additional data to clarify the mechanisms underlying this reduced fall risk, including the possibility that romosozumab might increase muscle mass. Furthermore, Chotiyarnwong et al. found that 6‐monthly subcutaneous denosumab reduced the number of fallers by approximately 20% in pooled data from five placebo‐controlled denosumab studies [68]. They concluded that these observations require further exploration and confirmation in studies with muscle function or falls as the primary outcome.

An emerging topic is the effect of anti-obesity drugs and bariatric surgery on osteoporosis, physical function, sarcopenia, and fall risk [69]. These treatments also are increasingly used by older persons, where loss of lean in addition to fat mass may predispose to sarcopenia and fall risk. On the other hand, treatment of obesity is beneficial and incretin-based drugs may offer specific benefits for bone structure. To be on the safe side, anti-obesity treatments require special attention for the maintenance of physical function and balance.

Furthermore, the International Classification of Diseases (ICD) is a standardized system for classifying diseases that supports data storage, retrieval, and analysis. The 11th revision, ICD-11 [70], adopted by the 72nd World Health Assembly in 2019 is currently being implemented by a number of countries. Using ICD-11 codes allows hip fractures to be more explicitly linked to the falls that caused them, including more options for more detailed contextual information, thereby enriching the data available for research. This creates opportunities for more granular epidemiological research, improved service evaluation, and enhanced monitoring of integrated care delivery.

One additional dimension that warrants explicit consideration is the role of emerging digital and monitoring technologies as enablers of a more coordinated approach to falls and fracture prevention. Wearable sensors enabling real-world assessment of balance and mobility, together with AI-driven predictive models, can enhance risk stratification by capturing ecologically valid signals that better reflect real-life fall risk and demonstrate superior predictive performance compared with traditional approaches [71, 72], while in silico clinical trials offer a complementary pathway to anticipate the effectiveness of preventive interventions and optimize their deployment at scale, by enabling simulation of virtual populations, prediction of intervention outcomes, and model-informed refinement of trial design and implementation [73, 74]. Beyond individual tools, these technologies create the conditions for integrating heterogeneous data streams (clinical, functional, and behavioral) into unified prevention pathways, supporting continuity of care across settings and improving alignment between fall risk detection and fracture prevention strategies. However, their implementation must be guided by robust evidence-generation frameworks addressing clinical validity, interpretability, usability in older populations, and long-term sustainability. Crucially, digital technologies should not be conceived as stand-alone solutions, but as enabling components within multidisciplinary, evidence-based care models, such as FLS and community-based prevention programs, where they can facilitate knowledge translation, support clinical decision-making, and inform policy development toward integrated, data-driven prevention strategies.

## Key components of fall and fracture prevention integration

To ensure the successful integration of fall and fracture prevention, it is vital to concentrate efforts on the following key areas:

- Integration of services for falls and fracture prevention should be a central priority.
- Future international guidelines, national protocols, and institutional care pathways should explicitly embed fracture prevention considerations within fall prevention strategies—and vice versa—to ensure comprehensive, bidirectional integration.
- Pre- and postgraduate educational and training initiatives of all healthcare professions should emphasize the integration of fall and fracture prevention, promoting a holistic approach rather than siloed management strategies.
- Interprofessional training should be strengthened to foster collaboration among healthcare professionals from different backgrounds, ensuring a coordinated, comprehensive approach to prevention, management, and treatment.
- Close collaboration between local, national and international organizations and societies focusing on fall and fracture prevention is essential to advance integrated fall and fracture prevention and the successful implementation of evidence-based practice.
- Integrated fall and fracture prevention must be recognized as a public health priority and prioritized by national and regional governments. Falls and fracture prevention should be incorporated into national healthy-ageing strategies as core interventions aligned with the WHO’s *Decade of Healthy Ageing* (2021–2030).
- Healthcare systems and policymakers should allocate adequate resources and funding for prevention, screening, interventions, and the implementation of integrated, interprofessional, intersectoral fall and fracture prevention and management care pathways that ensure continuity of care across clinical settings, including acute care, rehabilitation, primary care, and community services.
- There is a need to confirm the effectiveness and cost-effectiveness of integrated falls and fracture prevention strategies across clinical settings and populations.
- Falls and physical performance measures should be included as outcome measures in anti-osteoporosis medication trials.
- In terms of secondary prevention, more research is needed to understand the trajectory of fall risk across the entire lifespan, from age 50 years to the oldest old and to evaluate the effectiveness of secondary prevention strategies in this population.
- Emerging digital technologies, including wearable sensors, remote monitoring, and data-driven risk stratification, should be leveraged to support integrated fall and fracture prevention through continuous risk assessment and coordinated, personalized care pathways.

## Supplementary Information

Below is the link to the electronic supplementary material.

Supplementary file1 (DOCX 24 KB)
