# Supplementary material for: Position paper: a coordinated approach to fracture and fall prevention from seven international organizations
Source: Eur Geriatr Med. 2026 Sep 10;17(5):2407–22. doi: 10.1007/s41999-026-01596-7 (PMC13754987; doi:10.1007/s41999-026-01596-7)
Supplement: Supplementary file 1 [file 41999_2026_1596_MOESM1_ESM.docx]

**Position Paper: A Coordinated Approach to Fracture and Falls Prevention** from Seven International Organizations

**Supplementary Table 1**. **Participating organisations**

| **Organisation** | **Aim** | **Website** |
| --- | --- | --- |
| **EuGMS Special Interest Group on Falls and Fracture Prevention** | A multidisciplinary group within the European Geriatric Medicine Society connecting healthcare professionals and researchers across Europe to advance falls and fracture prevention research, best practice exchange, and implementation. | [https://www.eugms.org/groups-projects/falls-and-fractures](https://www.eugms.org/groups-projects/falls-and-fractures?utm_source=chatgpt.com) |
| **European Society for Clinical and Economic Aspects of Osteoporosis, Osteoarthritis and Musculoskeletal Diseases** | A non-profit organization fostering clinical science, economic evaluation and policy integration for optimal management of osteoporosis, osteoarthritis and related musculoskeletal disorders. | [https://www.esceo.org/](https://www.esceo.org/?utm_source=chatgpt.com) |
| **European Union of Medical Specialists- Geriatric Medicine Section** | Represents geriatricians in Europe, aiming to harmonize geriatric medicine training across Europe. | https://www.uemsgeriatricmedicine.org/ |
| **Fragility Fracture Network** | A global non-profit network working to improve care for people with fragility fractures by promoting multidisciplinary management, rehabilitation, secondary prevention, and policy change. | [https://fragilityfracturenetwork.org/](https://fragilityfracturenetwork.org/?utm_source=chatgpt.com) |
| **International Association of Gerontology and Geriatrics – European Region** | Promotes gerontological research, training and cooperation among member organizations with the goal of improving the well-being and quality of life of older people in Europe. | [https://iagg-er.eu/](https://iagg-er.eu/?utm_source=chatgpt.com) |
| **International Osteoporosis Foundation** | The world’s largest NGO dedicated to preventing osteoporosis and related musculoskeletal diseases, increasing awareness and improving diagnosis and treatment to prevent fragility fractures. | [https://www.osteoporosis.foundation/](https://www.osteoporosis.foundation/?utm_source=chatgpt.com) |
| **World Falls Prevention Society** | Promotes the multidisciplinary study, research dissemination, and implementation of fall prevention strategies in older adults and balance-impaired populations worldwide. | [https://worldfallssociety.org/](https://worldfallssociety.org/?utm_source=chatgpt.com) |
